# Reproductive skipping as an optimal life history strategy in the southern elephant seal, *Mirounga leonina*

**DOI:** 10.1002/ece3.4408

**Published:** 2018-07-30

**Authors:** Blaine D. Griffen

**Affiliations:** ^1^ Department of Biology Brigham Young University Provo Utah

**Keywords:** body mass, dynamic state variable model, individual heterogeneity, intermittent breeding, life history, lifetime reproductive output, marine mammal, reproductive skipping, stochastic dynamic programming, survival

## Abstract

Intermittent breeding by which organisms skip some current reproductive opportunities in order to enhance future reproductive success is a common life history trade‐off among long‐lived, iteroparous species. The southern elephant seal *Mirounga leonina* engages in intermediate breeding when body condition is low. While it is anticipated that this strategy may increase the lifetime reproductive output of this species, the conditions under which reproductive skipping are predicted to occur are not clear. Here I develop a dynamic state variable model based on published data that examines when southern elephant seals are predicted to optimally skip reproduction in order to maximize lifetime reproductive output as a function of current body mass, maternal age, and survivorship. I demonstrate that the optimal reproductive strategy for this species can include reproductive skipping, and that the conditions where this is optimal depend on patterns of mass‐dependent adult female survival. I further show that intermittent breeding can increase lifetime reproductive output, and that the magnitude of this benefit increases with the ability of individual animals to replenish depleted body mass through foraging. Finally, I show that when the environment is variable and foraging is reduced in bad years, the benefit of adopting an optimal strategy that includes reproductive skipping increases asymptotically with the frequency of bad years. These results highlight the importance of characterizing the pattern of adult survival in this species, as well as the need to identify other factors that may influence the prevalence and benefits of reproductive skipping.

## 1 INTRODUCTION

Reproduction is a central process for all organisms, and consequently, there is strong evolutionary pressure to optimize reproductive performance (Smith, 1978; Tuomi, Hakala, & Haukioja, 1983). For semelparous species that only reproduce a single time before death, this optimization results in maximum energy allocation to reproduction in order to produce the most offspring or the highest quality offspring possible (Pianka & Parker, 1975). For iteroparous species that are long‐lived and have the capability of reproducing multiple times throughout their lives, the optimum strategy may result in a range of reproductive possibilities (Pianka & Parker, 1975 and examples given therein). Because energy is limited, energetic trade‐offs in life history strategies often occur (Stearns, 1989), for instance, when the high cost of reproduction leads to reduced survival or lower reproduction in subsequent years. Consequently, organisms must balance the costs of current reproductive effort with future reproductive opportunities. One form of dealing with this trade‐off, that can sometimes be an optimal strategy, includes skipping current breeding opportunities (termed “intermittent breeding”) (Bull & Shine, 1979)—that is, the prudent parent hypothesis (Drent & Daan, 1980). Theory predicts that intermittent breeding is expected to occur as the cost of reproduction increases in terms of survival, energetic demand, or recovery time (Shaw & Levin, 2013).

Previous work demonstrates the link between intermittent breeding and several ecological and individual factors, including individual age (in fur seals: Beauplet, Barbraud, Dabin, Küssener, & Guinet, 2006; ibex: Rughetti, Demetteis, Meneguz, & Festa‐Bianchet, 2014; and seabirds: Zhang, Rebke, Becker, & Bauwuis, 2015), body condition (in birds: Calladine & Harris, 1997; Chastel, Weimerskirch, & Jouventin, 1995), previous reproductive experience (in birds: Desprez, Pradel, Cam, Monnat, & Gimenez, 2011; Pradel, Choquet, & Béchet, 2012), population density (in birds: Coulson, Duncan, & Thomas, 1982; and mountain goats: Hamel, Côté, & Festa‐Bianchet, 2010), and environmental conditions (in fish: Jørgensen & Fiksen, 2006). These factors, especially age and body condition, are linked with mortality risk, suggesting that mortality risk may play a particularly important role in determining when intermittent breeding should be beneficial. However, while the link between mortality risk following reproduction and intermittent breeding has been theoretically established (Shaw & Levin, 2013), the form of this relationship remains unclear. Additionally, while reproductive skipping has been associated with a range of individual, population, and environmental conditions across numerous types of organisms, the specific conditions where reproductive skipping is expected to occur have not been examined previously. This study presents an example of how these conditions can be determined for a specific system.

Southern elephant seals (*Mirounga leonina*) are relatively long‐lived capital breeders that were previously assumed to produce a single pup each year. However, recent studies at Marion Island (de Bruyn et al., 2011) and Macquarie Island (Desprez, Gimenez, McMahon, Hindell, & Harcourt, 2017) demonstrate that female elephant seals often skip breeding opportunities, and that this strategy leads to higher lifetime reproductive output than for females that breed annually, and that survival and probability of breeding are correlated (Desprez et al., 2017). However, reproductive skipping is not equal across all individuals, but is more common across poorer quality individuals (i.e., those that likely have decreased capability to replace lost energy stores and maintain good body condition) (Desprez et al., 2017). Despite the demonstration that intermittent breeding occurs in southern elephant seals, the factors that lead to intermittent breeding and the optimal strategy for this species remain unclear (McMahon, Harcourt, Burton, Daniel, & Hindell, 2017). The determination of when the optimal strategy is to breed vs. when it is to skip breeding may depend on adult state‐dependent survival, as reduced survival can strongly influence lifetime reproductive output (Curio, 1988; Shaw & Levin, 2013).

In southern elephant seals, breeding success (Arnbom, Fedak, & Rothery, 1994) and survival of offspring (McMahon, Burton, & Bester, 2000) depend on female body mass. Body mass varies seasonally due to the annual cycle of reproduction and molting (Carrick, Csordas, Ingham, & Keith, 1962), both of which incur large energetic costs (e.g., Boyd, Arnbom, & Fedak, 1993; McCann, Fedak, & Harwood, 1989), and may also vary with food availability (Carlini, Daneri, Marquez, Soave, & Poljak, 1997). It is also likely that adult female survival varies with body mass, although this has not been examined directly. Pistorius, Bester, Hofmeyr, Kirkman, and Taylor (2008) showed that survival of adult females during the postbreeding foraging trip was higher for more experienced (and thus likely larger) females than for first‐time breeders.

Finally, environmental predictability is one of the main drivers of life history evolution in general (Ferguson & Larivière, 2002), and of reproductive skipping in particular (Cubaynes, Doherty, Schreiber, & Gimenez, 2011; Shaw & Levin, 2013). In southern elephant seals, the number of seals breeding in any given year at Macquarie Island, for example, is influenced by environmental conditions (Van den Hoff et al., 2014), and reproductive skipping in this species is therefore expected to be closely associated with poor environmental conditions (Desprez et al., 2017). Poor environmental conditions may reflect periodic fluctuations associated with conditions such as El Niño, or directional shifts associated with climate change (Behrenfeld et al., 2006).

Here I use a dynamic state variable model to conceptually examine the optimal breeding strategy for southern elephant seals. Given that reproductive skipping has previously been documented in southern elephant seals (Desprez et al., 2017), I use this model to address five questions. First, does the optimal strategy for elephant seal lifetime reproductive output include instances of reproductive skipping? Second, could mass‐dependent adult female mortality risk influence the combination of maternal age and body mass where reproductive skipping may be optimal? Third, how might lifetime reproductive output be influenced by adopting a strategy that includes reproductive skipping as opposed to reproducing every year? Fourth, do the consequences of reproductive skipping on lifetime reproductive output for seals depend on their persistent individual quality or body condition (i.e., the “heterogeneity class” from Desprez et al., 2017)? Fifth, do the consequences of reproductive skipping on lifetime reproductive output for seals depend on annual variation in environmental quality and the frequency of bad years?

## 2 METHODS

I used dynamic state variable modeling to examine the conditions that are predicted to lead to reproductive skipping in southern elephant seals (Clark & Mangel, 2000; Houston & McNamara, 1999; Mangel & Clark, 1988). This technique has been successfully used to understand constraints and trade‐offs that underlie the dynamics of a large range of ecological systems (Mangel, 2015). Given the importance of body mass for reproductive success and for pup mass and survival in elephant seals, I used body mass as a proxy for fitness, and as the metric to be optimized.

I constrained body mass of adults within a maximum value (*x*_max_ = 1,060 kg) and a minimum value (*x*_crit_ = 140 kg). I chose these values based on the facts that females with larger mass produce larger pups (McCann et al., 1989), that body mass in postpartum females ranges from approximately 190–997 kg (Arnbom, Fedak, & Boyd, 1997; Arnbom et al., 1994; Postma, Bester, & de Bruyn, 2013a), and that body mass of pups at birth ranges from 28 to 60 kg (McCann et al., 1989). Mass of breeding females can vary substantially between sites (Postma, Bester, & de Bruyn, 2013b), but the value for *x*_max_ reflects the largest female (~1,000 kg postpartum) carrying the largest pup (60 kg), while the value for *x*_crit_ assumes that the smallest female departing from the breeding grounds (~190 kg, Postma et al., 2013a) could lose an additional 50 kg beyond this point before she has lost sufficient body fat and lean mass to succumb to death. While the starvation limit is not known for southern elephant seals, this value for *x*_crit_ is consistent with the minimum body mass for other marine mammals (Molnár, Klanjscek, Derocher, Obbard, & Lewis, 2009), scaled to the average length of an elephant seal in the age range examined here (Boyd, Arnbom, & Fedak, 1994).

The maximum breeding lifetime of southern elephant seals is from ages 3 to 23 (Arnbom et al., 1997; Hindell & Little, 1988), but functionally is often much shorter due to later maturity and/or earlier mortality (e.g., Carrick & Ingham, 1962; Carrick et al., 1962). I therefore examined a 10‐year breeding lifespan from ages 4 to 13 (i.e., backward iteration of the model started at age 14, where reproduction was assumed to be zero, and proceeded to age 4). The model developed here does not examine mass‐independent effects of age, such as senescence or higher costs of early reproduction (Desprez et al., 2017).

### 2.1 Model development

The processes of mass loss and gain are mass specific (i.e., larger seals often gain and lose more mass during a given activity). It is therefore necessary to perform model calculations for each biological process in the order that these processes occur, so that each mass change accounts for any preceding changes. I therefore began with female mass as she comes ashore to give birth and to breed (approx. September–October), followed by the postbreeding foraging period (approx. November–January), followed by the annual molt (approx. February–March), and finishing out each annual period with the postmolt foraging period (approx. April–August). Thus, if we are at year *y* in September, then by the next September we are at year *y *+* *1. The changes in mass occurring during each of these yearly time periods were therefore dependent on the mass changes that resulted from the biological activities in the preceding time periods. Female mass at the next time step [*x*(*y *+* *1)] is therefore a function of the arrival mass of the adult female (*x*) at the breeding site, the postpartum mass of the pup (*p*), the mass loss during nursing and breeding (*r*), the mass gain from postbreeding foraging during good and bad years ($f_{G_{b}}$ and $f_{B_{b}}$, respectively), the mass loss during molting (*m*_*l*_), and the mass gain from postmolt foraging during good and bad years ($f_{G_{m}}$ and $f_{B_{m}}$, respectively), each of which is expressed as kg. Combining these results in the following equation that gives the state dynamics:

(1) $$\begin{array}{c} x\left(y+1\right)=\left\{\begin{array}{c} \begin{array}{cc} x-p-r+f_{G_{b}}-m_{l}+f_{G_{m}}, & \text{with probability}\;(1-a)\\ x-p-r+f_{B_{b}}-m_{l}+f_{B_{m}}, & \text{with probability}\;(a) \end{array}, \end{array}\right. \end{array}$$

where *a* is the probability of a bad year. The derivation and calculation of each of these individual components is described below.

#### 2.1.1 Postpartum mass of the pup

Male elephant seal pups are generally larger than females, consequently, smaller adult females (<380 kg) generally give birth to female pups, while the ratio of male to female pups is similar for larger females (Arnbom et al., 1994). I therefore determined the postpartum mass of the pup using the following relationships given in Arnbom et al. (1994):

(2) $$\begin{array}{c} p=\left\{\begin{array}{c} \begin{array}{cc} 47.8\left(\pm 3.5\right)-107\left(\pm 49\right)\times e^{-0.0055(\pm 0.002)x}, & \text{for}\;x<380\\ 47.8\left(\pm 3.5\right)-107\left(\pm 49\right)\times e^{-0.0055(\pm 0.002)x}, & \text{for}\;x\geq 380\;\text{and}\;n>0.5\\ 52.6\left(\pm 7.2\right)-107\left(\pm 49\right)\times e^{-0.0055(\pm 0.002)x}, & \text{for}\;x\geq 380\;\text{and}\;n\leq 0.5 \end{array}, \end{array}\right. \end{array}$$

where *n* is a is a number between 0 and 1 drawn from a uniform distribution. Numbers in parentheses in this and subsequent equations indicate error in parameter estimation and were used in Monte Carlo simulations of the model.

#### 2.1.2 Mass loss during breeding

Mass loss during breeding has been reported in multiple studies and is the product of the mass lost per day (*r*_d_, kg/day) and the number of days (*d*_r_) required for lactation and depends on the initial mass of the mother (*m*_*i*_). An abundance of empirical data is available on these parameters (Arnbom et al., 1997; Carlini et al., 1997; McCann et al., 1989), and while reported mass losses in these studies were similar, I chose to use the following relationship from Carlini et al. (1997) from seals at Stranger Point, King George Island from 1994 to 1996, because it was based on the most data and had the highest *R* ^2^ value (*R* ^2^ = 0.77):

(3) $$r_{d}=0.013\left(\pm 0.001\right)\times m_{i}+2.48\left(\pm 0.91\right),$$

where *m*_*i*_ is initial postpartum mass of the mother. Carlini et al. (1997) also report the length of the lactation period as 22.1 ± 2.3 days (identical to the mean length reported by McCann et al., 1989). Further, the length of the lactation period appears to be constant regardless of breeding experience (Kirkman et al., 2004).

#### 2.1.3 Mass gain from postbreeding foraging

Foraging gains during both the postbreeding and the postmolting foraging periods may be estimated either as the product of the daily mass gain and the number of days spent foraging, or simply as the difference between pre‐ and postforaging mass measurements. However, while estimates of daily mass gain are available, none of these estimates account for the expectation that larger animals will gain more mass because they can consume more food per unit time (Le Boeuf et al., 2000). Bradshaw, Hindell, Sumner, and Michael (2004) provide estimates of relative mass gain (i.e., mass gain divided by departure mass), which does account for mass‐specific mass gain, during both the postbreeding and postmolting foraging periods. They showed that adult females gained 36.7% ± 3% of their preforaging body mass during postbreeding foraging:

(4a) $$f_{G_{b}}=0.367\left(\pm 0.03\right)\times m_{i},$$

where *m*_*i*_ in this case is the initial mass at the end of the breeding season (Table 1).

**Table 1:** Definition of model parameters

Model parameter	Symbol
Female mass	*x*
Pup mass at parturition	*p*
Female mass loss during breeding	*r*
Mass gain by breeders from postbreeding foraging during good foraging years	$f_{G_{b}}$
Mass gain by breeders from postbreeding foraging during bad foraging years	$f_{B_{b}}$
Mass gain by nonbreeders from postbreeding foraging during good foraging years	$f_{G_{b}}^{\prime }$
Mass gain by nonbreeders from postbreeding foraging during bad foraging years	$f_{B_{b}}^{\prime }$
Mass loss during molting	*m*_*l*_
Mass gain from postmolt foraging during good foraging years	$f_{G_{m}}$
Mass gain from postmolt foraging during bad foraging years	$f_{B_{m}}$
Daily mass loss during molting	*r*_d_
Number of days required to molt	*d*_r_
Pup mass at weaning	*m*_p_
Probability of pup surviving first year	*μ*_p_
Probability of maternal survival (annual)	*μ*_m_
Initial mass coming into a calculation^a^	*m*_*i*_

a *m*_*i*_ was used to represent the initial mass for several different calculations and its specific interpretation therefore depended on the context (described in each instance throughout the main text).

Mass gain from foraging during bad years will be less due to reduced food availability; however, the amount of reduction will vary across years and locations. Here I assume that mass gain from foraging in a bad year is 0.75 that from foraging in a good year, yielding:

(4b) $$f_{B_{b}}=0.75\times 0.367\left(\pm 0.03\right)\times m_{i}.$$

It is estimated that <1% of females that do not produce a pup in any given year will still return to shore to breed; rather, it appears that these females breed at sea while continuing to forage (de Bruyn et al., 2011; Desprez et al., 2017). These individuals are therefore expected to gain more weight given this extra feeding time. The length of the breeding period given above (22.1 days) is approximately 0.365× the length of the postbreeding foraging period, which is 60.5 ± 6.2 days (Carlini et al., 1997). I therefore assume that mass gain by nonbreeding females ($f_{G_{b}}^{\prime }$) will be 36.5% greater than for breeding females:

(4c) $$f_{G_{b}}^{\prime }=0.5\left(\pm 0.03\right)\times m_{i}$$

where *m*_*i*_ in this case is the initial mass coming into the breeding season. Analogously, mass gain while foraging by nonbreeding females during a bad year ($f_{B_{b}}^{\prime }$) is given by:

(4d) $$f_{B_{b}}^{\prime }=0.75\times 0.5\left(\pm 0.03\right)\times m_{i}.$$

#### 2.1.4 Mass loss from molting

Mass loss due to molting (*m*_*l*_) is determined by the number of days required to molt and the mass loss per day during the molt. The number of days required (*d*_m_) is an increasing function of the initial mass of the female (*m*_*i*_) and was determined from the data in table 2 of Boyd et al. (1993) to be (*R* ^2^ = 0.58):

(5) $$d_{m}=0.0396\left(\pm 0.008\right)\times m_{i}+1.92\left(\pm 4.05\right).$$

The combination of mass‐dependent duration of the molting period and the fact that nonbreeding individuals will gain more mass prior to the molt period than breeding individuals means that, all other things being equal, nonbreeding individuals should be expected to remain ashore longer during the molt.

Two estimates exist for the mass loss during any given day of molting. Boyd et al. (1993) estimated that adult females at Stromness Bay, South Georgia lose on average 4.7 kg/day, while Hindell, Slip, and Burton (1994) estimated that adult females at Macquarie Island lose 4.46 kg/day. Here I use the mean of these two estimates, or 4.58 ± 0.17 kg/day.

#### 2.1.5 Mass gain from postmolt foraging

I used the same reasoning and approach to determine mass gain after molting as I used for determining mass gain after breeding, described above. Bradshaw et al. (2004) provide estimates of relative mass gain during postmolt foraging (66.6% ± 3%), resulting in the following equations for mass gain from postmolt foraging during good years:

(6a) $$f_{G_{m}}=0.666\left(\pm 0.03\right)\times m_{i},$$

and during bad years:

(6b) $$f_{B_{m}}=0.75\times 0.666\left(\pm 0.03\right)\times m_{i}.$$

#### 2.1.6 Pup survival and body mass

In addition to changes in maternal body mass, the model also incorporates mortality estimates of the mother (*μ*_m_) and the pups (*μ*_p_), as well as pup body mass (*m*_p_). I assumed pup mortality to be mortality before the end of the first year of life. Oosthuizen, Altwegg, Nevoux, Bester, and de Bruyn (2017) followed 746 pups from birth onward between 1986 and 2016. They examined survival as a function of mass at weaning and found that survival increased with pup size; however, they were unable to determine whether this relationship was linear, saturating, or even unimodal. Postma et al. (2013a) also examined the influence of pup weaning mass on survival, dividing pup mass into 15‐kg intervals. I used WebPlotDigitizer to digitize the data in figure 6 from Postma et al. (2013a), from which I derived the following equation (insufficient information was given to enable determining standard deviations of parameter estimates, I therefore assume 5% variation):

(7) $$\begin{array}{c} 1-\mu_{p}=\left\{\begin{array}{c} \begin{array}{cc} 0.0 & \text{if}\;m_{p}<66\;\text{kg}\\ 0.14(\pm 0.007) & \text{if}\;m_{p}\geq 66\;\text{and}<80\;\text{kg}\\ 0.5(\pm 0.025) & i\;m_{p}\geq 80\;\text{and}<95\;\text{kg}\\ 0.67(\pm 0.0335) & \text{if}\;m_{p}\geq 95\;\text{and}<140\;\text{kg}\\ 0.93(\pm 0.0465) & \text{if}\;m_{p}\geq 140\;\text{and}<155\;\text{kg}\\ 1.0 & \text{if}\;m_{p}\geq 155\;\text{kg} \end{array} \end{array}\right. \end{array}$$

Pup mass at weaning (*m*_p_) is a function of initial maternal mass at parturition, as mass is transferred via nursing directly from the mother to the pup (McCann et al., 1989):

(8) $$m_{p}=0.171\left(\pm 0.05\right)\times m_{i}+31.457\left(\pm 24.3\right),$$

where *m*_*i*_ in this instance is initial maternal mass at parturition. Based on observed sizes of weaned pups, I capped *m*_p_ at 160 kg (McCann et al., 1989; Oosthuizen et al., 2017; Postma et al., 2013a).

#### 2.1.7 Maternal survival

**Figure 1:**
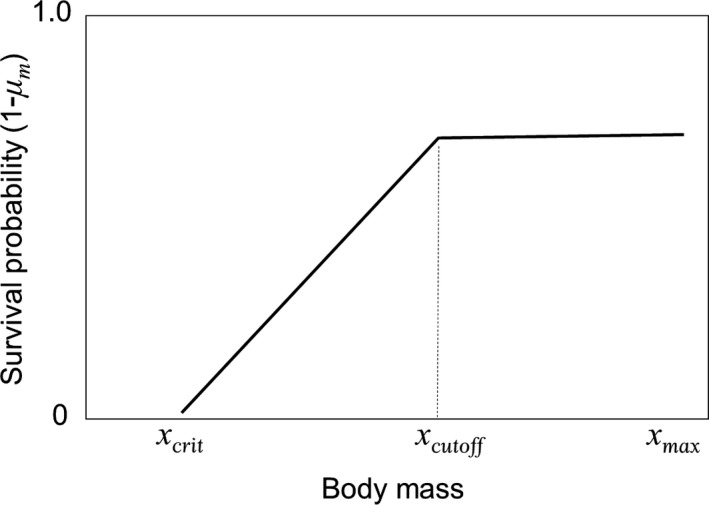
Conceptual strategy for mass‐dependent mortality used for maternal seals in the model. *x*_crit_ and *x*_max_ are as defined in the text. *x*_cutoff_ indicates the mass at which the maximum survival probability is reached. Any mortality that occurs for individuals with body masses above this point represent mass‐independent mortality

Adult female mortality (*μ*_m_) varies depending on year and location and can range from 0.05 to ~0.5 (Desprez et al., 2017; Pistorius et al., 2004). In addition to temporal and spatial variation, adult female mortality also differs for breeders and nonbreeders when body condition or the ability to replace depleted energy stores is poor, but not when body condition is good (Desprez et al., 2017). Differential survival for breeders and nonbreeders entered into the model via mass‐dependent maternal survival, as breeding greatly diminishes female body mass (e.g., McCann et al., 1989). In Figure 1, I demonstrate the conceptual strategy (i.e., a hockey stick model) of mass‐dependent survival incorporated here. Based on general concepts of animal physiology, I assume that if body mass falls below the minimum level (*x*_crit_) that mortality occurs. Above that critical threshold, I assume that survival probability increases linearly with body mass up to some maximum threshold (*x*_cutoff_), above which survival is mass independent. Mortality for individuals with body masses that exceed this threshold may occur from mass‐independent factors such as old age, ship strikes, or predation, for example. While this conceptual strategy is biologically reasonable, the maximum threshold for mass‐dependent mortality (*x*_cutoff_) in southern elephant seals is not known. Hence, I ran the model over a range of values for this threshold, from 140 kg (i.e., *x*_cutoff_ = *x*_crit_, or mass‐independent survival) to 500 kg, to determine how this threshold influences the optimal strategy for intermittent breeding.

#### 2.1.8 Putting it all together

There are two possibilities during any given year for a mature female—breed or do not breed. The fitness consequences of these two choices can be determined by combining the maternal body mass with the mass of the pup, as determined using the equations described above, each multiplied by their probability of survival. The fitness value of choosing to breed is therefore as follows:

(9a) $$V_{1}\left(x,y\right)=\left(1-\mu_{p}\right)m_{p}+\left(1-\mu_{m}\right)F\left\{\begin{array}{c} \left(1-a\right)\left(x-p-r+f_{G_{b}}-m_{l}+f_{G_{m}},y+1\right)\\ +a(x-p-r+f_{B_{b}}-m_{l}+f_{B_{m}},y+1) \end{array}\right\}.$$

While the fitness value of choosing not to breed is:

(9b) $$V_{2}\left(x,y\right)=\left(1-\mu_{m}\right)F\left\{\begin{array}{c} \left(1-a\right)\left(x+f_{G_{b}}^{\prime }-m_{l}+f_{G_{m}},y+1\right)\\ +a(x+f_{B_{b}}^{\prime }-m_{l}+f_{B_{m}},y+1) \end{array}\right\}.$$

The maximum expected production of pups that survive to age 1 between year *y* and year 14 for a female with mass *X*(*y*) = *x* is given as *F*(*x*,*y*), and the stochastic dynamic programming equation then becomes:

(10) $$F\left(x,y\right)=\text{max}\{V_{1}\left(x,y\right),V_{2}\left(x,y\right)\}.$$

I solved Equation (10) via backward iteration using stochastic dynamic programming in R, following Soetaert and Herman (2009), to determine the optimal breeding strategy for each combination of maternal age (ages 4–13) and body mass (from *x*_crit_ =* *140 to *x*_max_ = 1,060 kg, in intervals of 10 kg, using linear interpolation following Clark & Mangel, 2000). This backward iteration began at the end condition *F*(*x*,14) = 0.

### 2.2 Influence of *x*_cutoff_ and maximum survival

I examined whether varying the body mass where maximum survival is reached (*x*_cutoff_) influenced the model‐predicted conditions where reproductive skipping is the optimal choice. I ran the model with four different values for this cutoff: 200, 300, 400, and 500 kg. For each of these, I held the maximum survival constant at 0.912, the survival observed during postbreeding foraging for experienced breeders (Pistorius et al., 2008), and assumed no variation in environmental conditions (i.e., *a* = 0).

I similarly examined whether varying the maximum survival probability influenced the model‐predicted conditions where reproductive skipping is the optimal choice. I ran the model for six different values of maximum survival: 0.95, 0.85, 0.75, 0.65, 0.55, and 0.45. This encompasses the entire range of values for survival that have been reported for this species (Desprez et al., 2017; Pistorius et al., 2004). For each of these, I held the *x*_cutoff_ constant at 140 kg (i.e., equal to *x*_crit_, or conditions of mass‐independent survival), and assumed no variation in environmental conditions (i.e., *a* = 0).

### 2.3 Lifetime reproductive output with and without reproductive skipping

The model described above predicted the optimal reproductive strategy, or the strategy that is predicted to maximize lifetime reproductive output, given the relationships, assumptions, and constraints described above. I next performed Monte Carlo simulations using this model to assess the benefits to lifetime reproductive output of adopting this predicted optimal breeding strategy, relative to adopting a strategy of always breeding. The results of these two strategies converge if reproductive skipping is never the optimal strategy. Alternatively, if there are conditions where reproductive skipping is optimal, then we should expect higher lifetime reproductive success when the optimal strategy is followed rather than the strategy of annual breeding. I conducted simulations with *x*_cutoff_ set to 200, the maximum annual survival set to 0.912 (Pistorius et al., 2008), and assuming no variation in environmental conditions (i.e., *a* = 0). For each of the two breeding strategies (optimal breeding vs. annual breeding), I conducted 1000 simulations, returning the lifetime reproductive output, or the total number of offspring produced throughout life that survived to the end of the first year. In addition, the model returned the longevity of adult females, the mass at weaning of pups, and the number of reproductive attempts by each female (whether successful or not). Each of these latter three variables was used to mechanistically understand model results.

The error in each of the parameter estimates for each of the model equations was included in the simulations by drawing parameter estimates from normal distributions with means and errors given in the equations above. This allowed error from parameter uncertainty to propagate through the model. Each of the simulations was initiated with a 4‐year old female, with an initial body mass (*m*_*i*,_ kg) determined by the following equation from Arnbom et al. (1994):

(11) $$m_{i}=20.2\left(\pm 2.3\right)\times \text{age}+346\left(\pm 26.2\right).$$

### 2.4 Heterogeneity class

Desprez et al. (2017) showed that elephant seals can be separated into good and poor heterogeneity classes, which they hypothesized may reflect differences in the ability of seals to maintain body mass through foraging, and that these two classes subsequently show differences in the frequency of reproductive skipping. I mimicked heterogeneity class by altering the ability of seals to replace lost body mass while foraging following breeding ($f_{G_{b}}$ and $f_{B_{b}}$) and molting ($f_{G_{m}}$ and $f_{B_{m}}$). I took the existing range of possible values based on the mean and error values given above, and divided these regions into two separate parameter distributions, one that fell on the upper half and one that fell on the lower half of the original parameter distribution. Specifically, for the high quality heterogeneity class, I replaced Equations (4a), (4c), and (6a) above with Equations (12a), (12b), and (13), respectively:

(12a) $$f_{G_{b}}=0.4\left(\pm 0.015\right)\times m_{i}$$

(12b) $$f_{G_{b}}^{\prime }=0.55\left(\pm 0.015\right)\times m_{i}$$

(13) $$f_{G_{m}}=0.7\left(\pm 0.015\right)\times m_{i}$$

Similarly, for the low‐quality heterogeneity class, I replaced Equations (4a), (4c), and (6a), with (14a), (14b), and (15), respectively:

(14a) $$f_{G_{b}}=0.33\left(\pm 0.015\right)\times m_{i}$$

(14b) $$f_{G_{b}}^{\prime }=0.45\left(\pm 0.015\right)\times m_{i}$$

(15) $$f_{G_{m}}=0.63\left(\pm 0.015\right)\times m_{i}$$

I then repeated the model determination of the optimal strategies for reproductive skipping for both the good and poor heterogeneity classes and then repeated the Monte Carlo simulations for each of these two classes. From these simulations, I then compared the lifetime reproductive output and number of reproductive skipping events for seals of these two heterogeneity classes.

### 2.5 Frequency of bad years

I examined how the occurrence and frequency of bad years influenced the benefit of following an optimal reproductive strategy that includes skipping, compared to a strategy of trying to reproduce every year. To do this, I conducted Monte Carlo simulations across a range of probabilities that a bad year occurred (*a *=* *0, 0.2, 0.4, 0.6, 0.8, 1.0; *n* = 1,000 for each). For each of these simulations, I held maximum annual survival at 0.912, *x*_crit_ at 140 kg, and *x*_cutoff_ at 300. For each pair of simulations, I subtracted the lifetime reproductive output of the seal that followed the strategy of always reproducing from that of the seal that followed the optimal breeding strategy.

### 2.6 Statistics

Standard statistical tests are generally meaningless for results of simulation models where the possibility for replication is unlimited and the parameter values are already known to differ (White, Rassweiler, Samhouri, Stier, & White, 2014). I therefore did not conduct statistical comparisons of results of Monte Carlo simulations, but report only the effect size using Cohen's *d*, following McHuron, Costa, Schwarz, and Mangel (2017) and Pirotta et al. (2018) for analysis of model results.

## 3 RESULTS

### 3.1 Influence of *x*_cutoff_ and maximum survival

**Figure 2:**
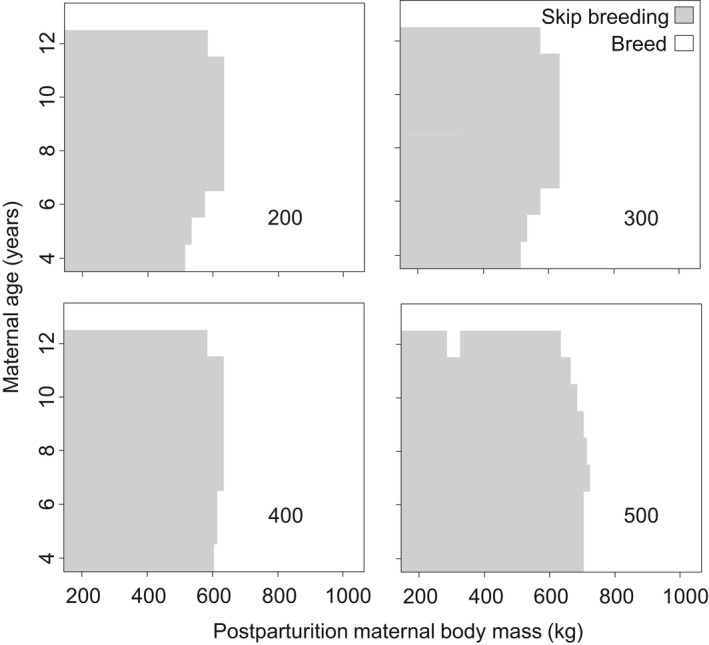
Model‐predicted combinations of postparturition body mass (*x*‐axis) and maternal ages (*y*‐axis) where the optimal strategy is to breed (white region) or to skip breeding in hopes of improving body condition and offspring quality in subsequent years (gray region). Numbers inside each plot show the body mass at which *x*_cutoff_ is reached. All results reflect model runs where the maximum survival was held constant at 0.912 with no environmental variation (*a* = 0)

**Figure 3:**
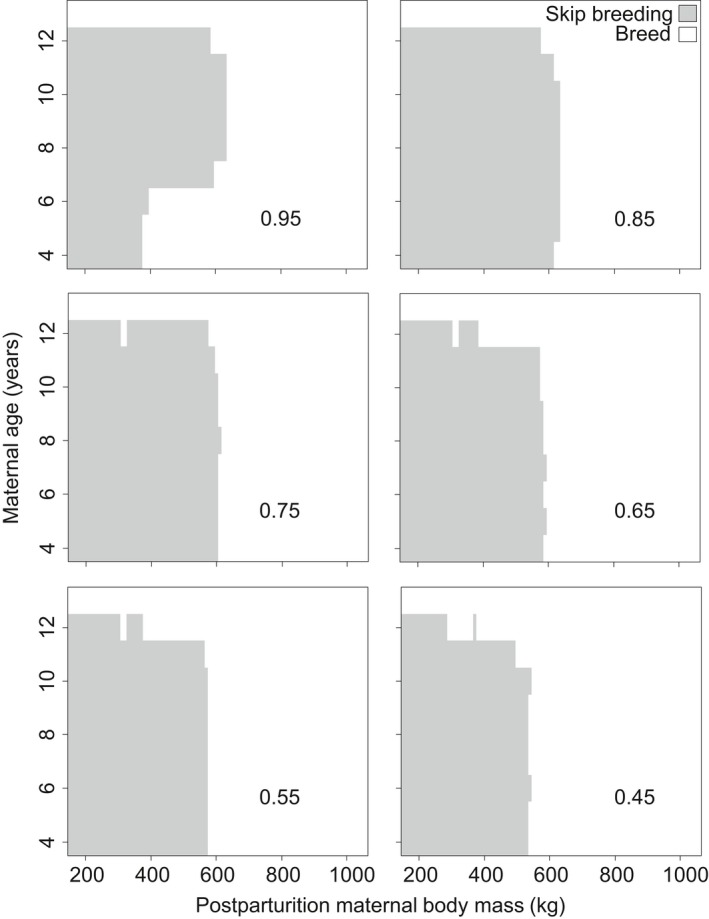
Model‐predicted combinations of postparturition body mass (*x*‐axis) and maternal ages (*y*‐axis) where the optimal strategy is to breed (white region) or to skip breeding in hopes of improving body condition and offspring quality in subsequent years (gray region). Numbers inside each plot show the maximum survival. The *x*_cutoff_ where this maximum survival was reached was held constant at 140 kg (i.e., mass‐independent survival) with no environmental variation (*a* = 0)

Consistent with previous field studies (de Bruyn et al., 2011), results of this dynamics state variable model predict that reproductive skipping in elephant seals is expected to be common. There were large areas of state space for maternal body mass and age where reproductive skipping is predicted to be the optimal strategy to maximize lifetime reproductive output. Reproductive skipping is generally optimal when body mass is low, although specific conditions vary with the cutoff body mass where maximum survival is reached (Figure 2) and with the maximum annual probability of survival (Figure 3). In general, the body mass where reproductive skipping is predicted to be optimal increases with the increase in *x*_cutoff_. While, except for at very high probabilities of survival (>0.90), the conditions for reproductive skipping are influenced relatively little by maximum survival probability.

### 3.2 Benefits of optimal breeding relative to annual breeding

**Figure 4:**
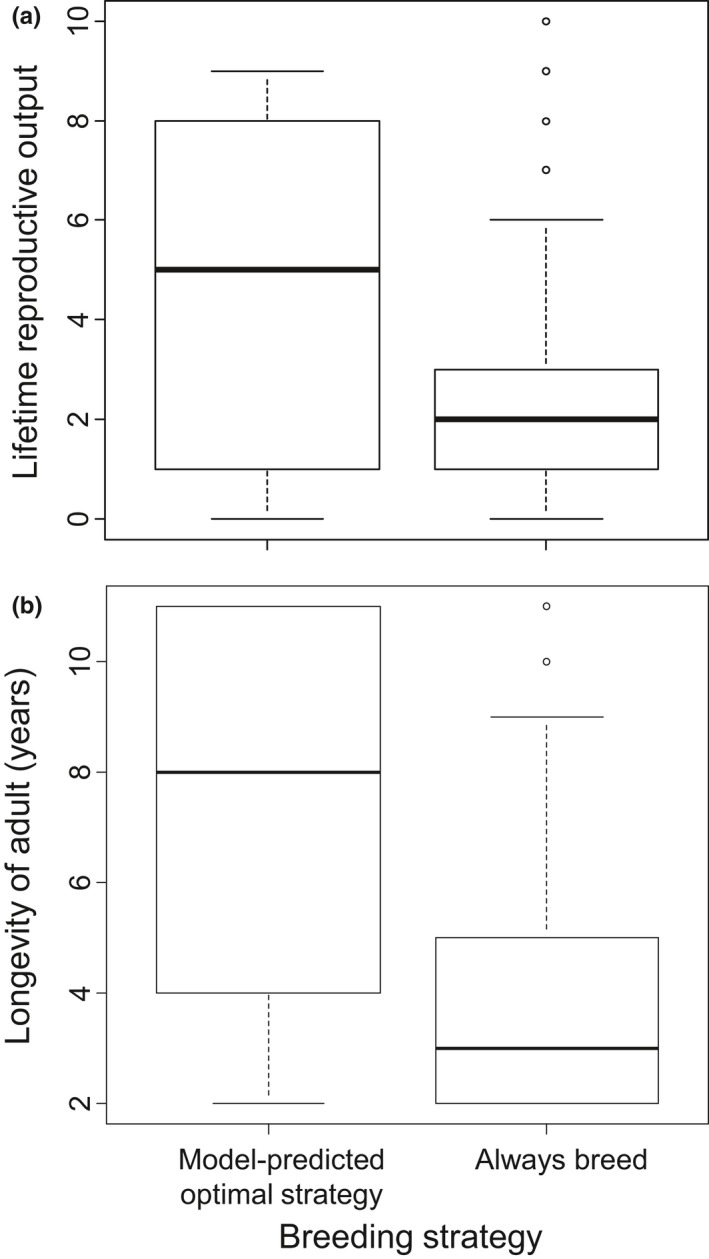
Comparison of lifetime reproductive output (a) and adult longevity (b) from 1,000 replicate Monte Carlo simulations with *x*_cutoff_ set to 200 kg and max annual survival set to 0.912 and no environmental variation (*a* = 0). Heavy line shows median value, box encompasses 25%–75% quartiles, whiskers encompass 95% of data, and circles fall outside this 95% range

Monte Carlo simulations of the model produced considerable variation in both adult female longevity and lifetime reproductive output, driven to a large extent by initial body mass at age 4 when the simulation was started. However, reproductive skipping also had a large impact, causing a trend toward higher lifetime reproductive output (Cohen's *d *=* *1.19, Figure 4a) and increased longevity (Cohen's *d *=* *1.21, Figure 4b). Higher lifetime reproductive output for the optimal breeding strategy was attributable to a combination of more reproductive attempts (possible because of greater maternal longevity) and larger body mass of pups (possible because of greater maternal body mass) that conveyed enhanced survival through the pup's first year of life. Specifically, females that followed the optimal breeding strategy had 5.5 ± 3.4 (mean ± *SD*) breeding attempts per female, with an average pup size at weening of 144.3 ± 27.7 kg. This was opposed to those that followed the annual reproductive strategy that had just 3.3 ± 2.7 reproductive attempts per female, with an average weening pup size of 126.4 ± 36.0 kg. When comparing these numbers of breeding attempts to the lifetime reproductive output shown in Figure 4, which shows only those pups that survived to age 1, it is clear that a greater proportion of pups died before age one from mothers who followed a strategy of breeding every year.

The number of reproductive skips varied across the 1000 simulations of the optimal reproductive strategy, with 133 individuals never skipping (i.e., given their body mass, the optimal strategy was to reproduce each year), 791 skipping once, and 76 skipping twice. By increasing the size at which maximum survival was achieved, the model produced instances with more than two skipping events.

### 3.3 Heterogeneity class

**Figure 5:**
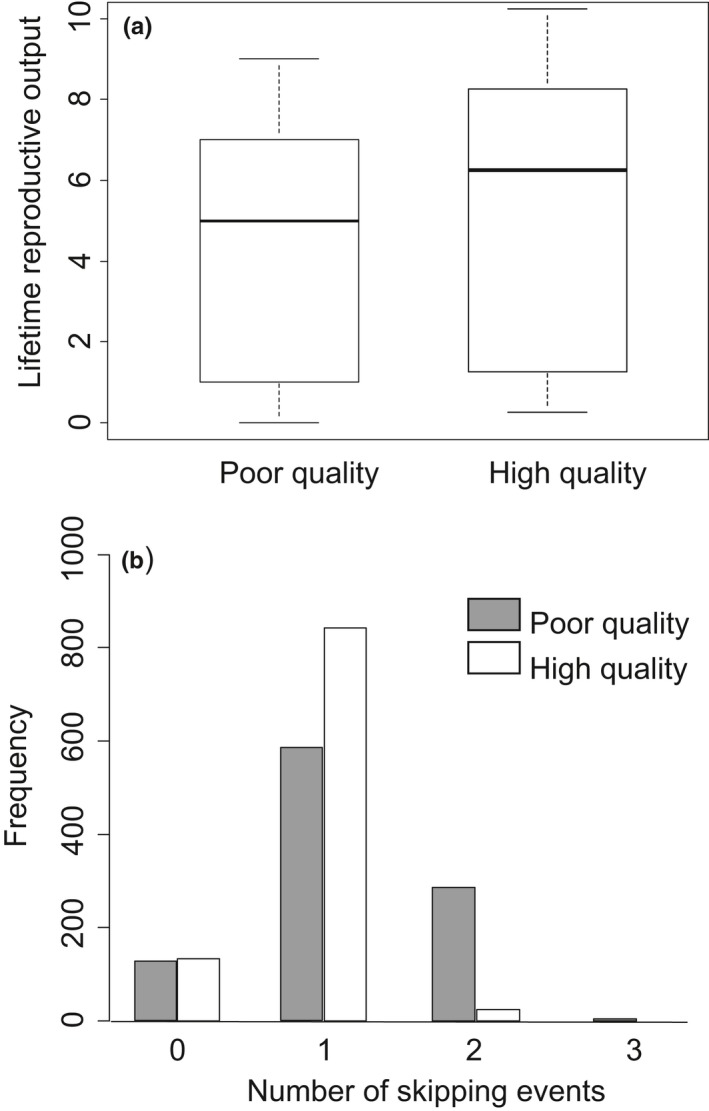
Differences in the lifetime reproductive output (a) and number of reproductive skipping events (b) for individuals of poor heterogeneity class and individuals of high heterogeneity class from 1,000 Monte Carlo simulations. Model parameters and boxplot description as given in Figure 4 legend

Consistent with previous results based on field capture–recapture studies (Desprez et al., 2017), reproductive skipping was slightly more prevalent among individuals of consistently poorer quality that had a reduced capability of replacing lost body stores (Cohen's *d *=* *0.11, Figure 5b). This resulted in lower lifetime reproductive output for these individuals, when following the model‐predicted optimal reproductive strategy, compared with higher quality individuals that were more capable of replacing lost energy stores, although the effect was not large (Cohen's *d *=* *0.07, Figure 5).

### 3.4 Frequency of bad years

**Figure 6:**
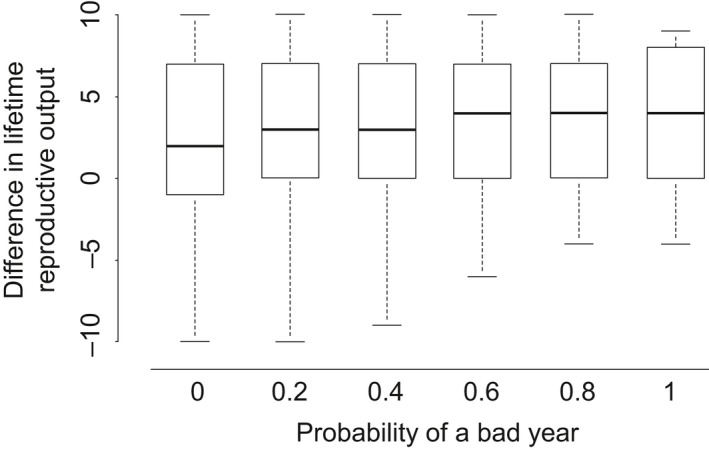
Benefit of following the optimal strategy based on the frequency of a bad year. *Y*‐axis shows the lifetime reproductive output of seals that follow the optimal strategy, minus the lifetime reproductive output of seals that attempt to breed every year. Positive (negative) values therefore indicate higher (lower) lifetime reproductive output for the optimal strategy compared to the strategy of annually attempting to breed. Each individual box plot shows results of 1,000 Monte Carlo simulations with a given probability of a bad year (*x*‐axis). Boxplot description as given in Figure 4 legend

The benefit of following the optimal strategy increased with the probability of a bad year (Figure 6), as reduced mass gain during bad years led to lower mass‐dependent maternal survival, smaller pups, and lower mass‐dependent pup survival. The benefit of following the optimal strategy increased from *a *=* *0 to *a *=* *0.6 and then remained constant with further increases in the frequency of a bad year.

## 4 DISCUSSION

### 4.1 Reproductive skipping as an optimal strategy

I have shown that the optimal reproductive strategy in southern elephant seals includes intermittent breeding. Based on the model parameterization and assumptions described above, seals in the Monte Carlo simulations skipped one or two breeding attempts throughout the 10‐year period examined, sometimes in succession, sometimes not. Other model parameterizations induced predicted instances of 3–5 skipping events during the same 10‐year period. Results of multiple skipping events are consistent with observed patterns in reproductive skipping for this species, where interruptions in breeding are common and seem to increase with age (de Bruyn et al., 2011). Thus, while organisms may not always behave optimally, the consistency between model‐predicted (this study) and observed patterns (de Bruyn et al., 2011) suggests that elephant seals may often employ optimal reproductive strategies by employing intermittent breeding. These patterns are also consistent with patterns of intermittent breeding in related species (northern elephant seals, Sydeman, Huber, Emslie, Ribic, & Nur, 1991), as well as in other mammals (e.g., Pilastro, Tavecchia, & Marin, 2003) and nonmammal species (e.g., Cubaynes et al., 2011).

### 4.2 Influence of mass‐dependent survival

I have shown that the conditions where reproductive skipping is the optimal strategy depend on patterns of adult female mass‐dependent survival. Previous estimates of adult female survival for this species demonstrate that it is spatially and temporally variable (Pistorius et al., 2004) and suggest that this variation is determined by food availability (Pistorius, Bester, & Kirkman, 1999). If survival is in fact determined by food availability, then this suggests that survival probability depends on body condition, as was assumed in the model formulation used here. Thus, survival probability is not only spatially and temporally variable, reflecting variation in environmental conditions, but is expected to also vary across individual animals. Studies in other large mammals demonstrate that body mass is an important determinant of adult survival (e.g., Festa‐Bianchet, Jorgenson, Bérubé, Portier, & Wishart, 1997; Loison, Langvatn, & Solberg, 1999). Results here highlight a need for more attention to the condition‐ or mass‐dependent mortality risk of adult female elephant seals in order to understand breeding strategies. Specifically, the model found that reproductive strategy, and thus lifetime reproductive output, is sensitive to the body mass where maximum survival is achieved (*x*_cutoff_ in the model here). Therefore, better understanding the influence of body mass on survival, and whether survival increases linearly with body mass above the lower critical threshold (*x*_crit_), as assumed here, could greatly improve our understanding of lifetime reproductive output, and thus the potential for population growth, for this species.

### 4.3 Mechanisms of increased lifetime reproductive output with reproductive skipping

In the model results presented here, I demonstrated that a reproductive strategy that includes intermittent breeding resulted in increased lifetime reproductive output compared to a strategy of annual reproduction. There were two mechanistic reasons for this increased lifetime reproductive output. First, intermittent breeding resulted in rapid gains in body mass that subsequently led to increased survival (because survival was assumed to be mass dependent). This in turn resulted in more overall breeding attempts. Second, larger maternal body masses due to reproductive skipping resulted in the production of larger pups that have higher likelihood of survival. Ultimately, the consequences of intermittent breeding for changes in female body mass will likely depend on environmental conditions, as previously predicted (Desprez et al., 2017), and as demonstrated using Monte Carlo simulation reported here.

Environmental conditions play a prominent role in elephant seal body mass (Oosthuizen, Bester, Altwegg, McIntyre, & De Bruyn, 2015), and one of the primary drivers of spatial and temporal variation in environmental conditions is El Niño, although its effects on southern elephant seals appear to be location specific (de Little, Bradshaw, Mcmahon, & Hindell, 2007; McMahon & Burton, 2005; Vergani, Stanganelli, & Bilenca, 2001). The frequency and intensity of El Niño is expected to increase with future climate change (Cai et al., 2014; Timmermann et al., 1999) and may therefore have important implications for optimal breeding by elephant seals.

### 4.4 Role of individual heterogeneity

I have shown that the benefits of reproductive skipping, in terms of gains in lifetime reproductive output, are higher for individuals that have a high ability to replenish lost body mass via foraging. In contrast, Monte Carlo simulations with the poorer class of seals (those that were less capable of replacing lost body mass) indicated that these individuals were forced to skip reproduction more frequently and thus had fewer offspring altogether. Thus, while reproductive skipping can increase the overall lifetime reproductive output, this benefit differs across individuals and is greatest for individuals with a high capacity for replacing lost energy stores in preparation for future reproductive events.

These model results may be used to inform the potential response of elephant seals to declining ocean conditions. I examined heterogeneity class by altering the rate of mass gain, showing that as this rate declines, reproductive skipping occurs more frequently, but has a weaker positive effect on lifetime reproductive output. Declining ocean productivity is expected to have identical results. With reduced ability to rebuild body mass in less productive oceans (because of reduced foraging success), reproductive skipping is expected to become more common, but will have diluted positive effects on lifetime reproductive output compared to periodic reproductive skipping in more productive oceans. Thus, changes in ocean productivity, whether because of changes in the frequency or intensity of El Niño events as described in the preceding section, or because of other environmental drivers, have implications for the optimal reproductive strategy of elephant seals.

Reproductive skipping is a common strategy across a diverse range of species (Bull & Shine, 1979), and determining when this strategy is expected to be employed is important fundamentally for understanding life history, and is important practically for accurately projecting population growth and dynamics. The modeling approach I have used here provides an example of how the expected conditions for reproductive skipping can be determined. I based this model on an abundance of empirical data available for southern elephant seals, thereby reducing the number of assumptions or extrapolations that were required. However, in systems that lack the abundance of empirical data available here, models can still be parameterized using known biological principles and trade‐offs (e.g., growth vs. reproduction) (Stearns, 1989), and by extrapolation from other systems that follow a similar life history strategy as the one being examined.

The model presented here focused on a single determinant of reproductive performance: body mass. Other factors are also known to be important determinants of mammal life history strategies. For instance, senescence in reproduction is common in female mammals (Packer, Tatar, & Collins, 1998). Senescence was implicitly included in the model here by only examining females during their reproductive prime (ages 4–13), while southern elephant seals are capable of breeding for an additional 10 years (Arnbom et al., 1997; Hindell & Little, 1988). While the model here could be further developed by explicitly including senescence or other factors known to influence reproductive life history in female mammals, the results here demonstrate the importance of intermittent breeding for lifetime reproductive success of this species, and point to likely patterns in reproductive skipping with changes in body mass. Ultimately, understanding the causes and consequences of intermittent breeding will be important for understanding and predicting changes in population growth rates of this species, especially under conditions of future environmental change.

## CONFLICT OF INTEREST

None declared.

## AUTHOR CONTRIBUTION

BD Griffen conceived the idea, designed the study, developed and implemented the model, analyzed the results, and wrote and edited the manuscript.

## DATA ACCESSIBILITY

Source code files of the models: Dryad https://doi.org/10.5061/dryad.1b8m917.
